# Identification and Quantification of Fumonisin A1, A2, and A3 in Corn by High-Resolution Liquid Chromatography-Orbitrap Mass Spectrometry

**DOI:** 10.3390/toxins7020582

**Published:** 2015-02-16

**Authors:** Masayoshi Tamura, Naoki Mochizuki, Yasushi Nagatomi, Koichi Harayama, Akira Toriba, Kazuichi Hayakawa

**Affiliations:** 1 Research Laboratories for Food Safety Chemistry, Asahi Group Holdings, Ltd., 1-21, Midori 1-chome, Moriya-shi, Ibaraki 302-0106, Japan; E-Mails: masayoshi.tamura@asahigroup-holdings.com (M.T.); yasushi.nagatomi@asahigroup-holdings.com (Y.N.); koichi.harayama@asahigroup-holdings.com (K.H.); 2 Graduate School of Medical Sciences, Kanazawa University, Kakuma-machi, Kanazawa-shi, Ishikawa 920-1192, Japan; 3 Research & Development Center, Asahi Group Holdings, Ltd., 1-21, Midori 1-chome, Moriya-shi, Ibaraki 302-0106, Japan; 4 Institute of Medical, Pharmaceutical and Health Sciences, Kanazawa University, Kakuma-machi, Kanazawa-shi, Ishikawa 920-1192, Japan; E-Mails: toriba@p.kanazawa-u.ac.jp (A.T.); hayakawa@p.kanazawa-u.ac.jp (K.H.)

**Keywords:** LC-Orbitrap MS, fumonisin A, corn, simultaneous analysis

## Abstract

Three compounds, hypothesized as fumonisin A1 (FA1), fumonisin A2 (FA2), and fumonisin A3 (FA3), were detected in a corn sample contaminated with mycotoxins by high-resolution liquid chromatography-Orbitrap mass spectrometry (LC-Orbitrap MS). One of them has been identified as FA1 synthesized by the acetylation of fumonisin B1 (FB1), and established a method for its quantification. Herein, we identified the two remaining compounds as FA2 and FA3, which were acetylated fumonisin B2 (FB2) and fumonisin B3 (FB3), respectively. Moreover, we examined a method for the simultaneous analysis of FA1, FA2, FA3, FB1, FB2, and FB3. The corn samples were prepared by extraction using a QuEChERS kit and purification using a multifunctional cartridge. The linearity, recovery, repeatability, limit of detection, and limit of quantification of the method were >0.99, 82.9%–104.6%, 3.7%–9.5%, 0.02–0.60 μg/kg, and 0.05–1.98 μg/kg, respectively. The simultaneous analysis of the six fumonisins revealed that FA1, FA2, and FA3 were present in all corn samples contaminated with FB1, FB2, and FB3. The results suggested that corn marketed for consumption can be considered as being contaminated with both the fumonisin B-series and with fumonisin A-series. This report presents the first identification and quantification of FA1, FA2, and FA3 in corn samples.

## 1. Introduction

**Figure 1:**
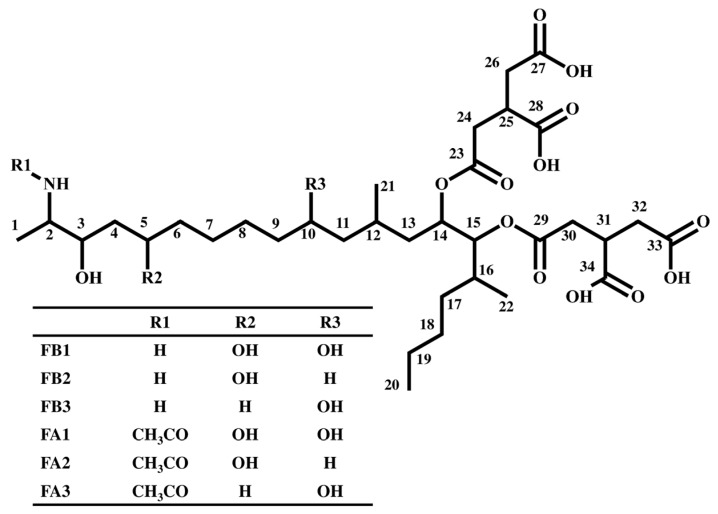
Chemical structure of fumonisins.

Fumonisins (Figure 1) are a type of mycotoxins produced by fungi of the *Fusarium* genus; several analogs have been discovered including the fumonisin A-series (FAs), fumonisin B-series (FBs), fumonisin C-series (FCs), and fumonisin P-series (FPs). Among these, FBs are known corn contaminants and constitute a major health risk as they may cause esophageal cancer in humans [1,2]. The detection of other analogs in *Fusarium* cultures has also been reported [3,4,5,6,7,8]; however, the extent to which they pose toxicity and contamination risks to food is unclear.

In previous reports, various fumonisin analogs were detected by quadrupole tandem mass spectrometry (MS/MS). Quadrupole MS/MS detects nominal mass owing to its low-resolution. The nominal mass has poor predictive power for unknown compounds due to the potentially numerous candidate chemical formulae. Consequently, there is little evidence that the compounds detected in such reports are fumonisin analogs. On the other hand, since high-resolution mass spectrometory (HRMS) including Orbitrap mass spectrometry (Orbitrap MS) and time-of-flight mass spectrometry (TOF MS), which enables high resolution analysis, provides an exact mass, it allows one to narrow down the candidate chemical formulae. Thus, it has superior predictive power in the identification of unknown compounds; it has been used to predict the structure of unknown mycotoxins [9,10,11,12,13,14].

We previously reported the detection of three compounds (compounds I, II and III) in a corn sample contaminated with mycotoxins (MTC-9999E) using liquid chromatography (LC)-Orbitrap MS [15]. In the study, because the candidate chemical formulae from measured exact masses suggested that an C_2_H_2_O group was added to fumonisin B1 (FB1), fumonisin B2 (FB2), and fumonisin B3 (FB3), the compounds were hypothesized to be fumonisin A1 (FA1), fumonisin A2 (FA2), and fumonisin A3 (FA3), which were the *N*-acetyl analogs of FBs. Hence, we identified compound I as FA1 by comparative analysis of FA1 synthesized via the acetylation of FB1. Moreover, an analytical method towards the quantification of FA1 was developed, and the concentration of FA1 in MTC-9999E was determined. Meanwhile, compounds II and III were predicted to be FA2 and FA3, respectively, owing to the fragmentation patterns of FB1 and FA1. However, there was insufficient evidence to confirm that the compounds were FA2 and FA3.

Thus, in the present study, FA2 and FA3 were synthesized by the acetylation of FB2 and FB3, and compounds II and III were identified. Additionally, we examined a method for the simultaneous analysis of the six fumonisins, namely FA1, FA2, FA3, FB1, FB2, and FB3, because their chemical structures are similar, they were considered to exhibit similar behaviors and toxicities. Moreover, the simultaneous analysis was used to quantify the contamination of FA1, FA2, and FA3 in corn samples contaminated with FB1, FB2, and FB3, and investigations regarding FA contamination in corn were carried out.

## 2. Results and Discussion

### 2.1. Syntheses of FA2 and FA3 and Identification of Compounds II and III in MTC-9999E

The acetylated derivative of FB2 (acetyl-FB2) was analyzed using LC-Orbitrap MS; a measured mass of 748.4120 was obtained, and the calculated formula was C_36_H_62_NO_15_^+^ with a theoretical mass of 748.4114, and a mass error of 0.77 ppm. FB2 and acetyl-FB2 were analyzed by NMR; a peak corresponding to the C-2 proton of FB2 was observed at 3.2 ppm, while the peak for the C-2 proton of acetyl-FB2 was observed at 3.9 ppm. This chemical shift was similar to those reported for FB1 and FA1, which is the acetylated form of FB1. In other words, the NMR results suggested that an *N*-acetyl group was bound to the C-2 of acetyl-FB2. Additionally, other chemical shifts (^1^H NMR (methanol-d_4_)) were observed at 0.790–1.010 (m, 9H), 1.139 (d, *J* = 0.012 Hz, 3H), 1.160–1.490 (m, 20H), 1.452–1.608 (m, 2H), 1.671 (brs, 1H), 1.959 (s, 3H), 2.473–2.819 (m, 8H), 3.120–3.220 (m, 2H), 3.747–3.820 (m, 2H), 3.850–3.925 (m, 1H), 5.181 (d, *J* = 0.021 Hz, 1H), and 5.349 (t, *J* = 0.008 Hz, 1H). As such, acetyl-FB2 was identified as FA2. Furthermore, the purity of FA2 was 60.4%.

In the same way, upon subjecting the acetylated derivative of FB3 (acetyl-FB3) to Orbitrap MS, the following data were obtained: a measured mass of 748.4122, calculated formula of C_36_H_62_NO_15_^+^, theoretical mass of 748.4114, and mass error of 1.03 ppm. In ^1^H NMR, the chemical shift of the C-2 proton appeared at 3.1 ppm for FB3 and 3.9 ppm for acetyl-FB3. Additional chemical shifts (^1^H NMR (methanol-d_4_)) were observed at 0.875–0.980 (m, 9H), 1.127 (d, *J* = 0.011 Hz, 3H), 1.160–1.520 (m, 20H), 1.650–1.750 (m, 2H), 1.834 (brs, 1H), 1.954 (s, 3H), 2.430–2.830 (m, 8H), 3.130–3.215 (m, 2H), 3.630–3.720 (m, 2H), 3.875–3.950 (m, 1H), 5.151 (td, *J* = 0.005, 0.018 Hz, 1H), and 5.349 (t, *J* = 0.008 Hz, 1H). As such, acetyl-FB3 was identified as FA3. The purity was 66.5%.

**Figure 2:**
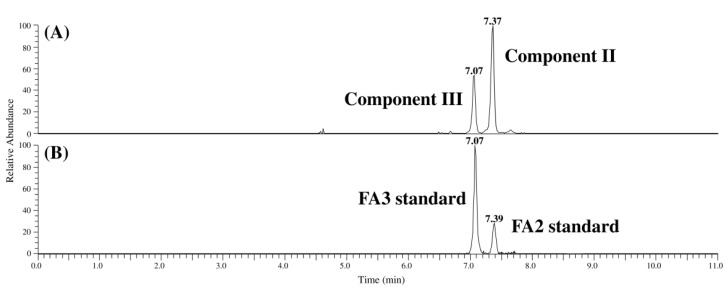
Chromatograms of compounds II, III, fumonisin A2 (FA2), and fumonisin A3 (FA3). (**A**) Chromatogram of compounds II and III; (**B**) chromatogram of FA2 and FA3.

**Figure 3:**
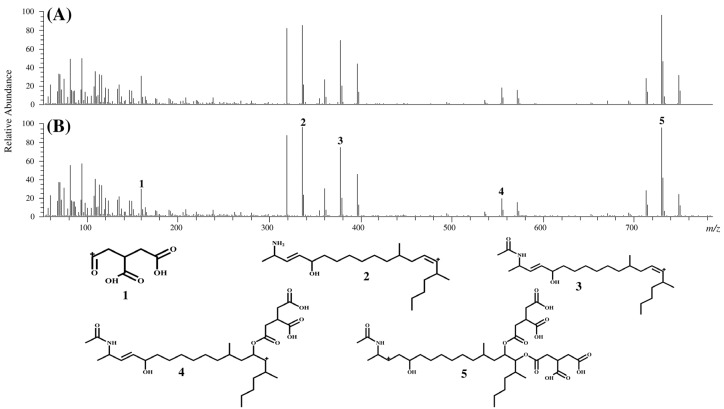
Product ion spectra of compound II and FA2, and characteristic peak assignment of FA2. (**A**) Product ion spectrum of compound II; (**B**) product ion spectrum of FA2.

**Figure 4:**
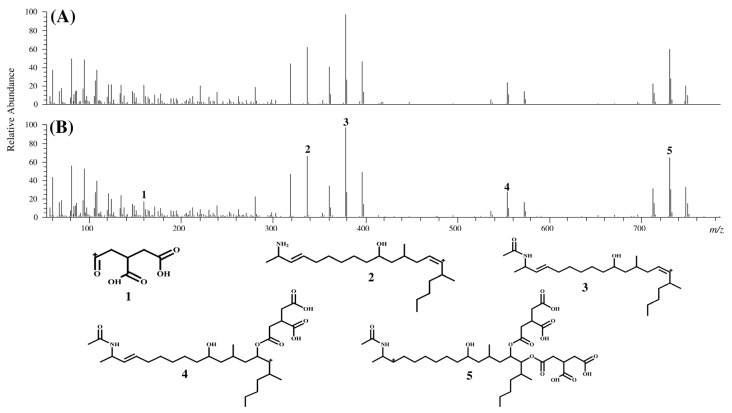
Product ion spectra of compound III and FA3, and characteristic peak assignment of FA3. (**A**) Product ion spectrum of compound III; (**B**) product ion spectrum of FA3.

The chromatograms and product ion spectra for compounds II and III in MTC-9999E as well as FA2 and FA3 obtained using LC-Orbitrap MS are shown in Figure 2, Figure 3 and Figure 4. The significant peaks in the spectra are labeled with identification (ID) numbers, corresponding to the numbers in Table 1 and Table 2, which summarized the measured mass, the theoretical mass, the calculated formula, and the mass error for each spectrum. Since the retention time and product ion spectra for compound II and FA2 as well as compound III and FA3 were in good agreement, compounds II and III were identified as the *N*-acetyl analogs of FB2 and FB3, namely FA2 and FA3, respectively.

**Table 1:** Characteristic peak assignment of the product ion spectrum of FA2.

ID	Measured mass (*m*/*z*)	Theoretical mass (*m*/*z*)	Calculated formula [M + H^+^]	Mass error (ppm)
1	159.0285	159.0288	C_6_H_7_O_5_	−1.77
2	336.3266	336.3261	C_22_H_42_NO	1.60
3	378.3369	378.3367	C_24_H_44_NO_2_	0.68
4	554.3661	554.3661	C_30_H_52_NO_8_	0.05
5	730.4014	730.4017	C_36_H_60_NO_14_	−0.32

**Table 2:** Characteristic peak assignment of the product ion spectrum of FA3.

ID	Measured mass (*m*/*z*)	Theoretical mass (*m*/*z*)	Calculated formula [M + H]^+^	Mass error (ppm)
1	159.0290	159.0288	C_6_H_7_O_5_	1.21
2	336.3259	336.3261	C_22_H_42_NO	−0.67
3	378.3366	378.3367	C_24_H_44_NO_2_	−0.12
4	554.3700	554.3687	C_30_H_52_NO_8_	2.26
5	730.4009	730.4008	C_36_H_60_NO_14_	0.06

Additionally, the characteristic product ion spectra of FAs, which suggested the cleavage of the tricarballylic acids, hydroxyl groups, and *N*-acetyl group were observed.

### 2.2. Validation of the Method

Extraction with a QuEChERS kit followed by purification using a Multistep 229 Ochra multifunctional cartridge was performed for the sample preparation. QuEChERS stands for quick, easy, cheap, effective, rugged, and safety, and serves as a preparation method for pesticide residues [16]. This method was used in the previously reported quantitative analysis of FB1, FB2, FB3, and FA1 [15], and thus was considered to be a viable method for the analysis of FA2 and FA3. The accuracy of the method in the determination of FA1, FA2, FA3, FB1, FB2, and FB3 was evaluated using the prepared corn sample. The results are shown in Table 3. The linearity, recovery, repeatability, limit of detection (LOD), and limit of quantification (LOQ) were acceptable at >0.99, 82.9%–104.6%, 3.7%–9.5%, 0.02–0.60 μg/kg, and 0.05–1.98 μg/kg, respectively. These results suggested that we successfully developed an acceptable method for the simultaneous analysis and quantification of FA1, FA2, FA3, FB1, FB2, and FB3 in corn.

**Table 3:** Performance of the method.

Validation Item	FA1	FA2	FA3	FB1	FB2	FB3
Linearity (*r*)	0.9996	0.9999	0.9993	0.9960	0.9946	0.9962
Recovery (%)	82.9	85.6	95.4	101.6	104.6	104.2
Repeatability (%)	2.7	9.5	6.5	5.3	3.7	7.1
LOD (μg/kg)	0.10	0.60	0.28	0.02	0.04	0.03
LOQ (μg/kg)	0.34	1.98	0.92	0.05	0.12	0.10

### 2.3. Quantification of FA1, FA2, FA3, FB1, FB2, and FB3 in Corn

The concentrations of FA1, FA2, FA3, FB1, FB2, and FB3 in corn samples were quantified via simultaneous analysis. MTC-9999E, MTC-9990, and FC-443, which were contaminated with fumonisins, were selected as the analytical samples. Because the individual concentrations of FB1 and FB2 in MTC-9999E exceeded the range of the calibration curves, they were diluted 10-fold. Additionally, seven samples of commercially available corn contaminated with FB1, FB2, and FB3 (C-1~7) were chosen, as reported previously [17]. The results are shown in Table 4. The analysis revealed that the 10 corn samples contaminated with FB1, FB2, and FB3 were also contaminated with FA1, FA2, and FA3.

**Table 4:** Concentration of fumonisins in corn samples.

Sample	Concentration of Fumonisins (μg/kg)					
	FA1	FA2	FA3	FB1	FB2	FB3
MTC-9999E	4177.7	4033.7	269.0	28633.2	8868.2	2033.2
MTC-9990	256.0	221.9	30.2	1231.8	320.2	189.3
FC-443	500.6	488.7	85.2	2661.0	714.8	358.0
C-1	62.5	45.0	30.6	660.5	114.6	53.4
C-2	10.6	6.6	2.6	309.4	37.2	19.8
C-3	2.7	2.8	2.0	90.4	21.3	11.5
C-4	42.4	23.4	8.7	461.8	86.4	52.1
C-5	59.7	84.2	23.4	1181.8	276.2	183.9
C-6	17.9	11.9	5.3	385.1	43.3	32.9
C-7	8.0	5.9	(<0.92)	150.9	16.4	12.2

In MTC-9999E, which contained the most FB1, FB2, and FB3, the FA contaminants were also observed at relatively high concentrations, namely 4177.7 μg/kg for FA1, 4033.7 μg/kg for FA2, and 269.0 μg/kg for FA3. Additionally, 8.0–62.5 μg/kg of FA1, 2.8–84.2 μg/kg of FA2, and (<0.92)–30.6 μg/kg of FA3 were also detected in commercially available corn. This result confirmed that samples contaminated with FBs were also contaminated with FAs. Because FAs are produced by *Fusarium moniliforme*, *F. verticillioides*, *F. proliferatum*, and *F. nygami* [3,4,5,6,7], the analyzed corn samples were likely contaminated with these fungi. Although there have been some previous reports regarding the detection of FAs in *Fusarium* cultures, this is the first report to describe the identification and quantification of FA1, FA2, and FA3 in corn samples.

## 3. Experimental Section

### 3.1. Sample, Chemicals, and Reagents

Mycotoxin reference materials (MTC-9999E, MTC-9990, and FC-443) obtained from Trilogy Analytical laboratory (Washington, DC, USA) were used as the corn samples naturally contaminated with fumonisins. The acceptance limits of FB1, FB2, and FB3 in the reference materials, with incorporated uncertainties, are shown in Table 5. Seven samples of corn were also obtained from local supermarkets in Japan in 2013.

**Table 5:** Acceptance limits of FB1, FB2, and FB3 in mycotoxin reference materials.

Sample	Acceptance Limit (mg/kg)		
	FB1	FB2	FB3
MTC-9999E	28.3 ± 7.6	7.1 ± 1.9	1.7 ± 0.5
MTC-9990	1.3 ± 0.3	0.2 ± 0.1	ND
FC-443	3.6 ± 1.4	0.8 ± 0.3	0.3 ± 0.1

FB1, FB2, and FB3 standards were obtained from Cayman Chemical Co. (Ann Arbor, MI, USA), LKT Laboratories, Inc. (St. Paul, MN, USA), and Medical Research Council (Swindon, Wiltshire, UK), respectively. Standard solutions containing 50 μg/mL of FB1, FB2, and FB3 in acetonitrile/water (1/1, *v*/*v*) were obtained from Romer Labs (Bukit Merah, Singapore). Methanol (LC/MS grade), acetonitrile (analytical grade), acetic acid (guaranteed reagent grade), ammonium acetate (analytical grade), dipotassium hydrogen phosphate (guaranteed reagent grade), *N,N*-dimethylformamide (guaranteed reagent grade), and acetic anhydride (guaranteed reagent grade) were purchased from Kanto Chemical Co., Inc. (Tokyo, Japan). Methanol-d_4_ (NMR grade) and Supelpak 2 were obtained from Merck KGaA (Darmstadt, Germany) and Sigma-Aldrich (Bellefonte, PA, USA), respectively. Water was purified using a Millipore (Molsheim, France) Milli-Q system. A Q-sep Q 110 QuEChERS extraction kit was obtained from RESTEK (Bellefonte, PA, USA). A MultiStep 229 Ochra multifunctional cartridge was obtained from Romer Labs (Bukit Merah, Singapore). A PTFE filter with a mesh size of 0.20 μm was acquired from Advantec Toyo Kaisha, Ltd. (Tokyo, Japan).

### 3.2. Sample Preparation

Sample preparation was carried out as described previously [15,17]. Specifically, a 2.5 g sample was placed in a 50 mL polypropylene centrifuge tube and 20 mL of 2% acetic acid aqueous solution/acetonitrile (1:1, *v*/*v*) was added. The samples were mixed at 250 rpm using a shaker (SR-2 DS; Taitec Saitama, Japan) for 1 h. The contents of the Q-sep Q110 were then added to the centrifuge tube. The mixture was vortexed for 20 s and centrifuged at 3000 rpm for 5 min. The supernatant (acetonitrile phase) was frozen at −30 °C for 1 h and was subsequently centrifuged at 3000 rpm for 5 min. Next, 5 mL of the supernatant, 1 mL of water, and 60 μL of acetic acid were mixed, and the mixture was added to the MultiStep 229 Ochra. The eluate (4 mL) was dried at 40 °C under a nitrogen stream and dissolved in 400 μL of 10 mM aqueous ammonium acetate/acetonitrile (85:15, *v*/*v*). Each sample was filtered using a 0.20 μm PTFE filter immediately prior to LC-Orbitrap MS analysis.

### 3.3. LC-Orbitrap MS Analysis

The LC-Orbitrap MS analysis was performed using an Ultimate 3000 system coupled to a Q-Exactive™ mass spectrometer (Thermo Fisher Scientific, Bremen, Germany). Xcalibur™ 2.2 software (Thermo Fisher Scientific) was used to control the instruments and process the data.

LC was performed using a 10 mM aqueous solution of ammonium acetate as solvent A and 2% acetic acid in methanol as solvent B [15,17]. The gradient profile was 2% B (0–2.0 min), 55% B (3.0–4.0 min), 70% B (4.1 min), 80% B (7.0 min), 95% B (7.01–8.0 min), and 2% B (8.01–11.0 min). The flow rate was set to 0.4 mL/min and the column temperature was maintained at 40 °C. The chromatographic separation was carried out using a Mastro C18 column (2.1 mm × 100 mm, 3 μm) obtained from Shimadzu GLC (Tokyo, Japan) with an injection volume of 5 μL.

The Q-Exactive™ mass spectrometer was operated in positive mode with a heated electrospray ionization source (HESI-II) and a spray voltage of 3.00 kV. The capillary and heater temperatures were 350 °C and 300 °C, respectively. The sheath gas and the auxiliary gas flow rates were 40 and 10 arbitrary units, respectively. The precursor ion scan was carried out in full MS mode at a resolution of 70,000 at an *m*/*z* value of 200 (3 scans/s), with an auto gain control (AGC) target of 3e6, a maximum injection time (IT) of 100 ms, and a scan range of 100–1000 *m/z*. The product ion scan was conducted in data-dependent MS^2^ mode (dd-MS^2^) using a resolution of 17,500 at an *m/z* value of 200, AGC target of 2e5, maximum IT of 200 ms, normalized collision energy (NCE) of 30 eV, stepped NCE of 50%, and scan range of 50–800 *m*/*z*.

### 3.4. Syntheses of FA2 and FA3 and Identification of Their Structures by NMR Analysis

FA2 was synthesized from FB2 as follows [15]: 2 mg of FB2 was weighed out in a 50 mL recovery flask and was dissolved in 0.1 mL of *N,N*-dimethylformamide. Subsequently, 0.7 mL of a 3 M aqueous solution of dipotassium hydrogen phosphate and 0.5 mL of acetic anhydride were added to the FB2 solution and stirred with a magnetic stirrer for 3 min. To the reaction mixture, 1.2 mL of water was added, and the solution was stirred for 10 min. To this solution, 20 mL of water was added and the entire volume of the reaction solution was loaded on Supelpak 2, previously filled in an open column. The column loaded with the reaction solution was washed four times with 15 mL of water and once with 20 mL of acetonitrile. The compounds were then extracted with 10 mL of acetonitrile and the extract was evaporated to obtain 0.29 mg of FA2. In the same way, 0.46 mg of FA3 was obtained from 2 mg of FB3. Each portion of FA2 and FA3 was dissolved in methanol-d_4_ and analyzed by NMR spectroscopy. ^1^H NMR (600 MHz) and HSQC spectra were recorded on a Bruker AV 600 instrument (Bruker, Karlsruhe, Germany). The chemical shifts were expressed in terms of δ (ppm) relative to the solvent signal (methanol-d_4_, δ^H^ 3.31, δ^C^ 49.0).

### 3.5. Validation of the Method

The method was validated by evaluating the linearity, recovery, and repeatability using a corn grit sample that contained 9.3 μg/kg of FB1 (FB2, FB3, FA1, FA2, and FA3 were not detected). The coefficient of linearity was determined from the calibration curves, which were constructed by plotting the peak areas of the prepared samples spiked with FA1, FA2, FA3, FB1, FB2, and FB3 against the concentrations of the analyte. The concentrations of FA1, FA2, FA3, FB1, FB2, and FB3 added to the samples were 5, 10, 50, 100, 500, 1000, and 5000 μg/kg. To the sample, 50 μg/kg of FA1, FA2, FA3, FB1, FB2, and FB3 were added for the recovery and repeatability evaluations. The repeatability was calculated by repeating the measurements six times on the same day and calculating the relative standard deviation of these measurements. The LODs and LOQs were defined as the concentrations that gave a signal to noise ratio (S/N) of 3:1 and 10:1, respectively.

## 4. Conclusions

The identification of two compounds, which were detected in a corn sample contaminated with mycotoxins (MTC-9999E), was performed using high-resolution LC-Orbitrap MS. Because the compounds were hypothesized to be FA2 and FA3, which were the *N*-acetyl analogs of FBs, FA2 and FA3 were synthesized by the acetylation of FB2 and FB3. By the comparative analysis of the retention time and product ion spectra of the detected compounds and synthesized FA2 and FA3, the compounds were confirmed to be the *N*-acetyl analogs of FB2 and FB3, namely FA2 and FA3.

A method for the simultaneous analysis of the six fumonisins, namely FA1, FA2, FA3, FB1, FB2, and FB3 was examined. The corn samples were prepared using a QuEChERS kit for extraction and a multifunctional cartridge for purification. The linearity, recovery, repeatability, limit of detection, and limit of quantification were determined to be >0.99, 82.9%–104.6%, 3.7%–9.5%, 0.02–0.60 μg/kg, and 0.05–1.98 μg/kg, respectively. As such, we successfully developed a viable method for the simultaneous analysis and quantification of FA1, FA2, FA3, FB1, FB2, and FB3 in corn.

Moreover, the simultaneous analysis of the six fumonisins revealed that the 10 corn samples contaminated with FB1, FB2, and FB3 were also contaminated with FA1, FA2, and FA3. Although there have been some previous reports regarding the detection of FAs in *Fusarium* cultures, this is the first report to describe the identification and quantification of FA1, FA2, and FA3 in corn samples. Based on the results of this study, corn marketed for consumption can be considered as being contaminated not just with FBs, but also with FAs. Since the link between the toxicity and mechanism of action of fumonisins has not been elucidated, it is necessary to conduct further investigations and research regarding fumonisins and their analogs.
